# Cardiac Cephalgia as a Manifestation of Acute Coronary Events: A Systematic Review

**DOI:** 10.7759/cureus.88398

**Published:** 2025-07-21

**Authors:** Rosemary Siby, Doju Cheriachan, Sanjay Kumar, Sara Khan, Heet N Desai, Srilakshmi K Jayaprakasan, Lubna Mohammed, Leslie Sangurima, Maujid Masood Malik, Nency Ganatra, Pousette Hamid

**Affiliations:** 1 Internal Medicine, California Institute of Behavioral Neurosciences & Psychology, Fairfield, USA; 2 Emergency Medicine, California Institute of Behavioral Neurosciences & Psychology, Fairfield, USA; 3 Internal Medicine, Bahria University and Dental College, PNS Shifa Hospital, Karachi, PAK; 4 Pediatrics, Dr B R Ambedkar Medical College and Hospital, Bengaluru, IND; 5 Biomedical Sciences, King Faisal University, Al Hofuf, SAU; 6 Neurology, California Institute of Behavioral Neurosciences & Psychology, Fairfield, USA

**Keywords:** cardiac cephalgia, coronary diseases, coronary events, headache, heart attack, heart failure, ischemic stroke, migraine

## Abstract

Acute coronary syndromes often have atypical presentations, especially in female and elderly populations, leading to considerable diagnostic challenges. Cardiac cephalgia (CC), or headache caused by myocardial ischemia, is a rare and often neglected manifestation, a situation that deserves the interest of medical professionals. This systematic review was conducted in accordance with the Preferred Reporting Items for Systematic reviews and Meta-Analyses (PRISMA) 2020 guidelines to synthesize available literature on this intriguing phenomenon. A thorough search was conducted on six databases: PubMed, SCOPUS, Web of Science, ProQuest, Google Scholar, and EBSCO, targeting papers published between 2000 and 2023. Ten peer-reviewed studies met the inclusion criteria, including randomized controlled trials, cohort studies, and case reports, with a total participant pool of 80,202. Several emergent themes were noted: the potential for CC to be the only symptom of ischemia, diagnostic misattributions to primary headache disorders, and clinical resolution noted following nitrate therapy or coronary interventions. Notwithstanding the narrow breadth of available evidence, which stems mainly from case reports, the findings suggest that clinicians must maintain a high index of suspicion for CC in patients who present with unexplained headaches and cardiovascular risk factors. Contemporary clinical guidelines rarely recognize this presentation, and additional prospective studies are necessary to develop diagnostic criteria and incorporate cardiac investigations into headache evaluation protocols.

## Introduction and background

Headache disorders are among the most common neurological complaints in clinical practice, which have conventionally been divided into primary headaches, including migraine and tension-type headaches, and secondary headaches caused by some underlying pathological processes. Cardiac cephalgia (CC) is one of the most severely under-recognized causes of secondary headache in which myocardial ischemia directly causes headache as opposed to the classic anginal symptoms and can cause diagnostic errors with lethal results.

First reported by Lipton et al. in 1997, CC is a condition in which patients report headache during myocardial ischemia episodes in the absence of the traditional cardiac symptoms of chest pain, palpitations, or dyspnea that clinicians often rely upon to indicate the presence of coronary pathology [1].

The lack of classical cardiac symptoms poses a significant problem in terms of diagnosis since patients are commonly misclassified with primary headache disorders in the emergency departments or neurology clinics, leading to unnecessary treatments like analgesics, triptans, or NSAIDs and late identification of acute coronary syndrome (ACS). Such diagnostic neglect has dire consequences, such as the elevated risk of ventricular arrhythmias, heart failure, or sudden cardiac death. The pathophysiology is not fully understood; however, current hypotheses suggest that referred pain mechanisms are involved, with shared spinal cord pathways between cardiac afferent nerves and cranial pain pathways. This systematic review aims to synthesize the existing evidence on CC as a manifestation of ACS, including its clinical characteristics, diagnostic features, and treatment options, to enhance clinical awareness and improve patient outcomes.

This systematic review addresses three critical knowledge gaps: What are the clinical features that are reliable in differentiating CC from primary headache disorders? How does early coronary intervention affect headache resolution and cardiac outcomes? What is the diagnostic pathway that optimizes detection without unnecessary testing?

The hypothesis is that CC is a distinct clinical condition that requires (a) independent diagnostic criteria founded on both neurological and cardiac presentation and (b) urgent coronary evaluation in high-risk individuals with new-onset exertional headache. The research presents evidence-based guidelines for clinicians who encounter this diagnostic dilemma at the neurology-cardiology interface [1].

Literature review

Overview of the Studies

The systematic review yielded 10 papers, comprising seven case reports and three observational studies conducted in various countries and settings. Altogether, these studies have contributed to the development of a general understanding of the clinical characteristics, diagnostic approaches, and treatment patterns observed in patients with CC. This is most apparent since most of the included studies were conducted on middle-aged to older adults with risk factors for coronary artery disease (CAD), including hypertension, diabetes, smoking, hyperlipidemia, and a family history of CAD.

Clinical Presentation of CC and Response to Anti-anginal Therapy

The most frequent presentation of CC in the various studies was encountered as exertional or stress-related headaches. Such headaches are typically characterized as pressing, dull, or throbbing and primarily affect the occipital, bifrontal, or vertex areas of the head.

Although not officially used in the majority of research, the clinical characteristics of cardiac cephalalgia - namely, the onset of headache with effort, the absence of neurologic symptoms, and resolution with cardiac treatment - meet the International Classification of Headache Disorders 3rd Edition (ICHD-3) criteria for secondary headache attributed to a disorder of homeostasis or cardiac ischemia. A common trend observed was that it was not accompanied by chest pain, and this was among the causes of delay in the diagnosis of the condition [2-4]. Essentially, a majority of the patients consulted neurologists with chief complaints of primary headache disorders like migraine and were misdiagnosed and managed as primary headache disorders before cardiac evaluation [5]. Although this uniformity enhances diagnostic clarity, the majority of studies, such as those by Bini et al. [6] and Chowdhury et al. [7], are case-based and lack standardized diagnostic criteria.

Another variable that was evident in all the works was the stabilization of headache symptoms once anti-anginal therapy, especially nitrates, was administered. Some case reports described immediate headache improvement that occurred after the use of sublingual nitroglycerin, providing support for the idea that cardiac ischemia is a significant factor in the development of such headaches [8]. Furthermore, it is also important to note that therapeutic evidence was obtained from the percutaneous coronary intervention (PCI), which helps alleviate recurrent headache episodes in patients identified with coronary stenosis.

Diagnostic Approaches

A few of the frequently used diagnostic procedures included ECG, stress test, coronary angiography, and cardiac biomarkers to establish the cardiac manifestation of such symptoms [9]. In one case, it was observed that cardiac investigations should be included in the assessment algorithm for patients presenting with a headache that lacks a clear history and etiology, with risk factors for cardiovascular disease. Few studies provide a framework for differentiating CC from primary headaches based on objective criteria. Moreover, there is an apparent gap in guidelines for neurologists encountering atypical headache presentations, guidelines that would aid in the timely referral to cardiologists.

Specific Manifestations

Other studies established that CC presented with specific sex-specific differences, particularly in women. The women reported less symptomatic chest pain; instead of common chest pain, palpitations and fatigue were reported in most of the female patients. Research indicates that while both men and women commonly report chest discomfort during ACSs, women are less likely to present with classic symptoms such as chest pain and sweating. Instead, they more frequently experience nausea, vomiting, shoulder or back pain, and shortness of breath [1]. Unfortunately, this variation in symptom presentation can lead to delays in seeking medical attention, as women may not recognize their symptoms as indicative of ACS. This goes a long way in showing why a healthcare practitioner should consider gender perception while assessing a patient with an unusual headache symptom.

For instance, research showed that migraine with aura in women increased the propensity of major cardiovascular events, including myocardial infarction and ischemic stroke [10]. Patients with migraine have a high risk of developing ischemic stroke, especially younger women with both migraine and aura [11].

Headache as the only symptom for myocardial ischemia is not common, and only a few cases have been reported in the literature on CC [12]. Although research indicates a vascular overlap between migraine and cardiovascular disease, they do not provide a direct causal link to CC. This correlates with a cross-sectional study implemented with a focus on Danish people involving more than 50,000 participants, and they realized a connection between migraine and myocardial infarction, atrial fibrillation, and venous thromboembolism [13]. The study, while large in scope, examines broader cardiovascular risks associated with migraines rather than specifically targeting CC, thus limiting its specificity.

In another of their findings, it was established that the severity of migraine in the patients who had ischemic events was higher than that in the patients without such conditions [14]. That is why Migraine Disability Assessment Scale (MIDAS) scores were significantly increased in cardiac patients, providing evidence to support the assumption that cardiac headaches are characterized by a distinct clinical picture. However, since the study examined MIDAS scores retrospectively, the results could be influenced by recall bias.

In addition, from another perspective, researchers have examined cortical hypoperfusion in patients with headaches and discussed the direct ischemic cause of headaches in acute cardiac diseases [15]. The authors recommended that systemic hypoperfusion may alter cerebral autoregulation in some manner and lead to referred pain in the form of a headache.

Headaches due to myocardial ischemia are more often expressed as nonprimary headaches and do not require typical symptoms such as photophobia, nausea, or visual disturbances, which are characteristic of the primary headache [16]. These are mostly secondary and generally do not localize to the frontal area, but rather to the occipital or parietal area, which is more likely to occur while one is engaged in an activity or during stress. These observations collectively suggest that CC may be part of the vasogenic spectrum of cerebrovascular and cardiovascular diseases. Systemic inflammation, endothelial dysfunction, and platelet activation, which have been established as playing a part in migraine as well as ischemic heart disease, strengthen this concept.

Diagnosis of CC is challenging since it is atypical and has a highly variable presentation. An included randomized controlled trial (RCT) reported that CC findings indicate it can be the sole manifestation of myocardial ischemia in up to 27% of patients, and it can be exertional. Routine investigations, such as ECG, cardiac enzymes, and stress tests, may yield normal results, and coronary angiography may be necessary to confirm the diagnosis [14]. The headache can be migraine-like or mimic tension-type headaches and lacks any characteristic features. Pain location is variable, and there is typically no history of headache. The most significant diagnostic clues are late onset, exertional provocation, and vascular risk factors. Awareness is low since headaches are not typically recognized as a symptom of cardiac events.

Therefore, incorporating cardiac-indicating health screening for patients presenting with new-onset or atypical migraines has the potential to enhance the detection of cardiovascular diseases in these patients. This lends support to the argument that neurologists and cardiologists should attend to patients with high-risk headache presentations.

Clinical Implications

Although CC is a rare and inconclusive diagnosis, the reviewed literature stresses the increased need for consideration in clinical settings. The findings of the study help in advocating for the early diagnosis of these patients, thus minimizing unnecessary neurological procedures and addressing any possible acute coronary event. Therefore, CC may be underreported rather than rare, which warrants attention from neurologists and cardiologists. Besides these 10 papers, data found in the literature supplements and affirms the foregoing conclusions and hypotheses on the link between CC and ACSs. Migraine, and especially migraine with aura, has been linked to cardiovascular risk in several epidemiological and cohort studies.

## Review

Methods

Inclusion and Exclusion Criteria

The inclusion criteria were primary research studies, including RCTs, cohort studies, observational studies, and case reports, that involved adult patients (18 years and older) presenting with ACSs. The studies had to assess headache as a primary presenting symptom with objective cardiac confirmation, which was defined as diagnostic evidence of myocardial ischemia based on ECG changes, elevation of cardiac biomarkers (e.g., troponins), or findings from coronary angiography. Furthermore, studies that described headache as a significant or comorbid aspect of acute coronary events were included, provided they were published in peer-reviewed journals and available as full text in the English language. While the primary focus was on original research, one systematic review and one narrative review were also included due to their synthesis of rare clinical cases not otherwise captured in the primary literature.

Articles without original data, such as letters, editorials, reviews (with noted exceptions), or opinion pieces, were excluded. Studies were also excluded if objective confirmation of ACS was lacking or if the headache was mentioned only as a secondary or incidental symptom. Investigations involving non-cardiac populations, or where the headache was unrelated to myocardial ischemia, were not considered. Non-English studies were excluded due to the unavailability of translation resources, which may introduce language bias. Additionally, studies not available in full text were excluded to maintain consistency in data extraction and quality assessment.

Study Selection and Screening

The search results were all imported into a reference management system, and duplicate records were systematically deleted to avoid bias during the selection process. Initial screening of titles and abstracts to determine relevance was performed by two of the authors (RS and DC), with discrepancies resolved through discussion or consultation with a third author (SK) as needed. The studies that passed the first criteria were read in full text, and their relevance to CC, objectivity, and sufficient clinical description were considered to ensure the quality and applicability of the evidence included.

Data Extraction and Quality Assessment

In this case, data were collected by using a data extraction form that was adapted to have the following variables: authors of the study, the year of the study, country, study type, sample size, clinical setting, diagnostic technique and tools, the type of intervention measures, and the outcome of the study. According to the Case Report Guidelines (CARE) checklist, the quality of all the case reports was assessed. Newcastle-Ottawa Scale (NOS) checklists were used for evaluating observational research, while Consolidated Standards of Reporting Trials (CONSORT) checklists were used for other types of research, such as analytical ones.

Synthesis Strategy

Since the types selected by the review included studies that varied in their design features and types of outcomes, a narrative synthesis was used to synthesize the data. Accordingly, the discussion of the study and how themes were grouped was based on categories such as clinical presentation of children, diagnostic features considered in the study, response to treatment, and future practice.

Results

A total of 10 peer-reviewed journal articles published from 2016 to 2022 were found. These studies were selected for their relevance to differentiating CC from primary headaches, evaluating diagnostic methods, and assessing the impact of coronary interventions on headache resolution. They were consistent with the research question by offering clinical, diagnostic, and interventional information essential to defining and treating CC.

Of the research reviewed, six studies were quantitatively driven [13,15-19], including two cohort studies [13,16] and two cross-sectional studies [17,18]. Additionally, two studies were categorized as RCTs [2,14], while two were classified as case reports [12,20]. Additionally, while the primary focus was given to original research, one systematic review [20] and one narrative review [15] were included due to their synthesis of rare clinical cases that were otherwise unrepresented in the primary literature. These reviews, not being considered primary data sources, were included to gain contextual knowledge.

The total number of subjects (n = 80,202) is primarily drawn from large cohort and cross-sectional studies. Specifically, Adelborg et al. [13] contributed a total of 51,032 subjects, while Siak et al. [17] and Kurth et al. [18] contributed 402 and 27,858 subjects, respectively. The case reports [12,20] each included a single patient. This variation in sample size explains the vast numerical difference compared to the number of studies performed.

Quality was evaluated with the eight-item Joanna Briggs Institute (JBI) checklist. Studies were scored independently by three reviewers (RS, DC, and SK), and consensus scores were reached through discussion to resolve discrepancies. Scores could range from 0 to 8. Scores <4 indicated lower quality (greater risk of bias), and scores ≥4 indicated higher quality (lower risk of bias). No threshold score was applied to exclude studies; instead, quality ratings were used to inform interpretation, but not to determine eligibility.

**Figure 1:**
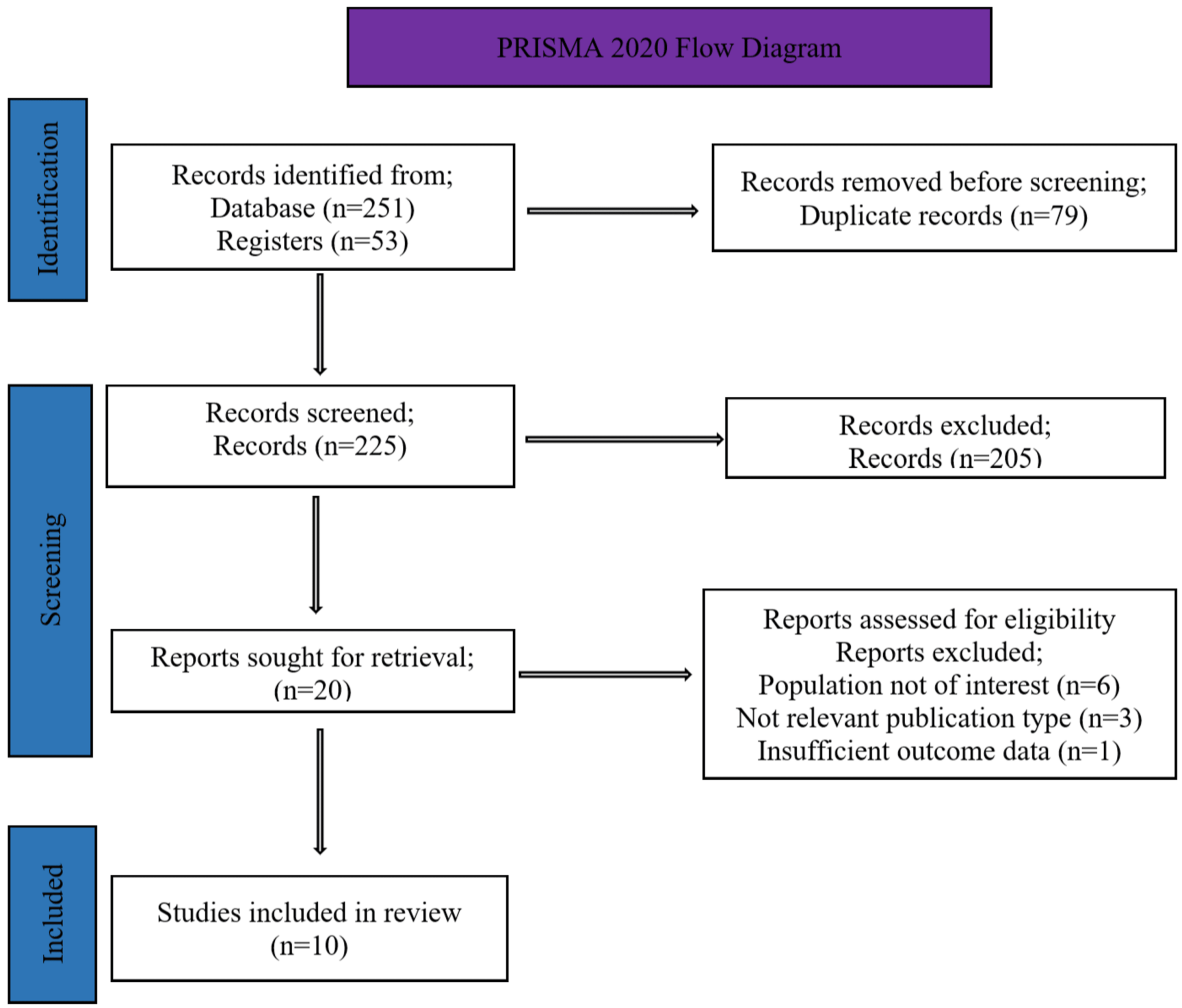
PRISMA 2020 flow diagram showing study identification, screening, eligibility, and inclusion process PRISMA, Preferred Reporting Items for Systematic reviews and Meta-Analyses

We found 304 records from the database and registry searches (251 and 53, respectively). Following the removal of 79 duplicates, 225 records were title- and abstract-screened. Of these, 205 were excluded. Two reviewers independently screened the full-text reports of the remaining 20. Discrepancies were resolved by consensus, with input from the third reviewer where necessary. A total of 10 studies were included, as indicated in the Preferred Reporting Items for Systematic reviews and Meta-Analyses (PRISMA) 2020 flow diagram (Figure 1).

Quality Assessment

To establish the rigor of this systematic review, we subjected the included sources to a thorough quality assessment. In line with this, the three investigators (RS, DC, and SK) used the eight-question JBI Tool for all the included studies. The risk of bias in every study was examined, and every journal article was given a score between <4, indicating “low quality” and high risk for bias. On the other hand, scores of >4 were assigned to articles that have a high quality and low risk of bias, as summarized in Table 1.

**Table 1:** Quality assessment of the included studies

Author	Risk of bias	Quality
Gül et al. [3]	Low	High
Chowdhury et al. [12]	High	Low
Adelborg et al. [13]	Low	High
Hassan et al. [14]	Low	High
Navarro-Pérez et al. [15]	Low	High
Kok et al. [16]	Low	High
Siak et al. [17]	High	Low
Kurth et al. [18]	High	Low
Sun et al. [19]	High	Low
Wang et al. [20]	Low	High

Table 2 provides a detailed summary of the characteristics of each study, including aspects such as author, country of origin, research design, setting, sample size, measures, and outcomes. The table serves as a replacement for the complete data extraction form. 

**Table 2:** Characteristics of the included studies The key variables extracted — including study design, clinical focus, cardiac diagnostic measures, and main headache-related outcomes — are summarized here. CC, cardiac cephalgia; RCT, randomized controlled trial

Author	Country	Design	Setting	Sample	Measure	Outcome
Gül et al. [3]	N/A	RCT	Hospital	100	Migraine and red blood cell distribution width	Patients with migraine have inflammation in the cardiovascular system.
Chowdhury et al. [12]	Bangladesh	Case study	N/A	1	Headache	Occurrence of unusual headaches is a symptom of coronary events.
Adelborg et al. [13]	Denmark	Cohort study	General population	51,032	Migraine and cardiovascular disease	Exhibition of migraine increases the risk of cardiovascular diseases.
Hassan et al. [14]	Egypt	RCT	Hospital	200	Migraine and ischemic stroke	The development of migraine has been associated with an increased risk of coronary events and ischemic stroke.
Navarro-Pérez et al. [15]	N/A	Narrative review	PubMed	82 cases	Characteristic of CC	Headaches with migraine are symptoms of CC.
Kok et al. [16]	USA	Cohort study	Hospital	585	Migraine and coronary heart disease	Headaches with migraine increase the occurrence of coronary diseases.
Siak et al. [17]	N/A	Cross-sectional	Hospital	402	Relationship between coronary functioning and migraine	There is a positive correlation between migraine and coronary dysfunction
Kurth et al. [18]	US	Cross-sectional	Hospital database	27,858	Migraine with aura and cardiovascular diseases	The presence of migraine with aura increases the likelihood of developing cardiovascular diseases.
Sun et al. [19]	China	Case report	N/A	1	CC and myocardial infarction	The occurrence of CC increases the occurrence of acute myocardial infarction.
Wang et al. [20]	China	Systematic review	Online medical databases	32 articles	Cortical hypoperfusion in headache and CC	Headaches with cortical hypoperfusion increase the risk of CC.

Notably, no scoring cutoffs were used to exclude studies from the review. The quality ratings were used descriptively to inform the interpretation of the findings, rather than being used as a criterion for inclusion or exclusion.

Discussion

ACS has a highly variable clinical presentation with extremely variable features, such that when the atypical symptoms occur, the diagnosis cannot be predicted. Instead of classic chest pain, patients present in a non-classic fashion with symptoms such as shortness of breath, back or abdominal pain, throat pain, or even toothache. Of all these atypical presentations, exertional headache due to isolated cardiac ischemia is rare, and case reports of it are infrequent [21]. Nevertheless, these presentations, otherwise known as CC, cannot be ignored, particularly among persons who have atherosclerotic risk factors. In the second case, headache during exertion may be a predictor of silent myocardial ischemia, and immediate cardiac investigation is reasonable, regardless of the headache's benign character.

The overall evidence in the systematic review is convincing and shows that CC is a clinically significant yet potentially underacknowledged manifestation of acute myocardial ischemia. The 10 studies synthesized point to three critical themes with essential implications for clinical practice: diagnostic misinterpretation, the responsiveness of therapies to anti-anginal interventions, and the necessity of interdisciplinary collaboration.

Therapeutic Responsiveness and Diagnostic Implications

The improvement of headache symptoms after the application of nitrates or PCI was uniformly noticed as the most remarkable finding in several case series. This response, however, was not consistently reported across all cases, and the use of therapeutic response as a diagnostic marker needs to be interpreted with caution. No study was placebo-controlled, and outcomes were not compared with non-responders. This course of therapy is the main distinction between CC and primary headache disorders, which should not improve with cardiac medications [22]. The vascular etiology hypothesis is primarily supported by the rapid alleviation of symptoms with the vasodilator effect of nitroglycerin in many case reports in the literature, suggesting that this phenomenon may be due to compromised myocardial perfusion, which in turn causes cerebrovascular changes manifested as cephalgia.

The anecdotal improvements relied upon, that is, the immediate improvement after the administration of nitroglycerin, should, however, be interpreted with caution. However, this by itself is not enough to diagnose CC. That responsiveness in such therapy is suggestive but not conclusive, as it is not controlled by comparison with other groups or placebo-controlled trials, which provide more convincing evidence. Additionally, alternative explanations and comorbid neurological diseases are rarely discussed in these reports, which may make it confusing when attributing symptoms, which generates a risk of over-diagnosing CC based on treatment reaction alone. Still, this observation raises helpful questions regarding the pathophysiological processes that are under-researched in the accessible literature.

Diagnostic Problems and Clinical Presentation

CC is not easy to diagnose because it is atypical, and its presentation is very variable. In the narrative review by Navarro-Pérez et al. [15], CC was the only symptom reported in as many as 27% of cases, according to pooled case data. This percentage, however, comes from uncontrolled case reports, and 'sufferers' is a term applied to those patients who were diagnosed retrospectively. One challenge is that patients can have normal ECG and enzyme results on routine cardiac testing, which can lead to misclassification. In these cases, adjunctive imaging or risk-stratification tools can be indicated. ECG, cardiac enzymes, and stress tests may be routine, but coronary angiography must be performed to confirm it [23].

The headache may be of a migraine type or resemble tension-type headaches, and it lacks any defining characteristics. This phenotypic overlap makes diagnosis more difficult. Clinicians must be vigilant for red flags, including exertional onset, a lack of headache history, and cardiovascular risk factors - characteristics not typically seen in primary headache disorders. The pain has a variable location, and a history of headaches is usually absent. Late-onset exertional provocation and vascular risk factors are the most critical diagnostic leads. The level of awareness is poor because headache is not generally considered a symptom of cardiac events. Among the studies included, the only narrative review, by Navarro-Pérez et al. [15], evaluated CC using the ICHD-3 diagnostic classification. None of the original studies used formal diagnostic criteria for CC.

It is a significant patient safety concern, and it has been evident in many studies that CC is misdiagnosed as a primary headache disorder. There are clinical overlaps between CC, migraine, and tension-type headaches that lead to diagnostic uncertainty, which, in most instances, results in a delay in appropriate cardiac evaluation and management [23]. This diagnostic delay is particularly problematic because untreated ACS can progress to cause fatal outcomes such as lethal arrhythmias, heart failure, and sudden cardiac death. The information suggests that exertional or stress headaches in patients with cardiovascular risk factors should trigger an emergent cardiac workup rather than a standard neurologic diagnosis [24]. However, existing clinical guidelines do not adequately address this diagnostic challenge, resulting in a significant gap in evidence-based practice recommendations.

Across the included studies, shared diagnostic features included exertional or stress-provoked headache, absence of focal neurologic signs, improvement of symptoms with nitrates, and concomitant cardiovascular risk factors. ECG abnormalities, raised cardiac biomarkers, and coronary angiography results were uniformly utilized to establish the diagnosis of myocardial ischemia. While no study has formally suggested a diagnostic algorithm, these investigative and clinical patterns can help generate suspicion of CC in unusual presentations of headache.

Gender Presentation Differences

The point of significant gender differences in the presentation and diagnosis of CC has been brought up in several studies. Women are more likely to present with isolated headache signs and symptoms that are not accompanied by chest pain, which is the foundation of preferential referrals to neurology and delays in cardiac evaluation. This fact is consistent with the current state of literature that indicates that CAD develops in women unusually, and current diagnostic procedures fail to take into account this and other gender-related peculiarities. The unacknowledged CC in women can add to the documented gender disparities in outcomes of coronary syndromes, which creates an immediate need for gender-sensitive clinical practice guidelines and provider education programs. These observations were derived from general impressions across the included studies and were not the result of pre-specified subgroup analyses. Since subgroup analyses were not performed, these gender findings are exploratory. Sex-stratified analyses should be included in future research to clarify diagnostic differences.

Knowledge Gaps and Pathophysiological Mechanisms

The pathophysiology of this phenomenon had not been well established. However, some studies have attempted to establish possible correlations between the localization of headaches and the territories of the coronary arteries. Further neurophysiological studies are needed to investigate the proposed mechanism of referred pain transmission via overlapping spinal cord connections between cardiac afferent fibers and cranial pain pathways [25]. This hypothesis is primarily based on theoretical models and extrapolated from animal studies; however, direct neurophysiological evidence in humans remains limited. Additionally, the role of systemic hypoperfusion and inflammatory mediators, as well as the autonomic dysfunction in the pathogenesis of CC, is primarily theoretical. It represents significant gaps in knowledge that limit the understanding of this condition.

Interdisciplinary Collaboration Implications and Clinical Practice

The existing evidence consistently indicates that the successful diagnosis and treatment of CC can only be achieved when professionals specializing in cardiology, neurology, and emergency medicine collaborate closely. However, the current health systems lack consistency in the management of patients with suspected CC, which leads to care fragmentation and misdiagnosis. A systematic screening protocol for cardiovascular investigation in high-risk patients with unexplained headaches could improve the accuracy of diagnosis and patient outcomes [26]. The cost efficiency and effectiveness of such methods, however, remain topics for future investigation. Economic analyses, prospective cohort studies, imaging techniques, and autonomic function testing represent promising areas for future research. Incorporation of validated headache screening instruments into protocols for cardiovascular risk evaluation could also enhance interdisciplinary diagnostic workflows.

The consequences of such results necessitate fundamental changes in clinical practice approaches for evaluating headaches. CC remains high on the list of suspicions that clinicians should uphold when dealing with patients who have cardiovascular risk factors and present with exertional headaches [27]. A therapeutic and diagnostic strategy, as exemplified by a therapeutic trial of nitrates, can be effective but requires standardization of protocols for these interventions.

It, though potentially increasing diagnostic suspicion, also risks hypotension and lacks standardization of protocol and interpretation. Further studies are needed to establish the safety and efficacy of this approach in this specific situation. Dominguez et al. suggested that educational programs on CC should be introduced among emergency physicians, neurologists, and primary care providers to improve condition recognition and adequate treatment [28]. An update of clinical guidelines that includes CC as a differential diagnosis of headache disorders would result in a significant reduction of diagnostic delay and potentially improve patient outcomes substantially.

Limitations and Recommendations for Future Studies

CC has now been receiving some level of attention and public attention metrics, yet several questions about this condition remain unanswered, for instance, its prevalence, diagnostic criteria, and prognosis. Further studies are needed to develop a uniform diagnostic response assessment system for CC, possibly including clinical characteristics (e.g., exertional headache with no focal neurology), biomarker findings (e.g., troponin rise), angiographic demonstration, and symptomatic response to cardiac treatments.

Additionally, evidence-based diagnostic strategies may involve the systematic use of ECG, stress testing, cardiac biomarkers, and coronary imaging. While none of the studies included functional imaging techniques like PET, fMRI, or SPECT, their theoretical applicability in detecting cerebral hypoperfusion or changed autonomic activity in CC makes them worthy of exploratory investigation. They could clarify neurovascular mechanisms and distinguish cardiac-origin headache from primary conditions.

The findings of this review point to several areas for future research and highlight significant implications for clinical practice. CC remains an underdiagnosed and poorly understood clinical phenomenon. For enhanced recognition and management, future research should focus on refining diagnostic criteria, elucidating underlying mechanisms, and directing standardized care pathways. There are currently no established diagnostic criteria for CC. Nevertheless, drawing on the observations from this review, we suggest a possible clinical strategy that can help shape a diagnostic scheme. These include headache with onset related to exertion or emotional stress, the absence of focal neurological deficits, the presence of cardiovascular risk factors or a history of ischemic heart disease, a lack of response to conventional headache medications, and objective documentation of myocardial ischemia on ECG, cardiac biomarkers, or coronary angiography.

Although case reports provide essential clinical information, they are formally limited by small sample sizes, the absence of controls, and potential publication bias. Hence, any conclusions based on such sources must be skeptical and confirmed by larger observational or controlled research.

Additionally, the findings of this review could be influenced by publication bias, particularly due to the inclusion of rare case reports that are more likely to be published when the results are either unusual or positive. Furthermore, the absence of significant prospective studies limits the generalizability and strength of the conclusions drawn.

First, future large-scale studies are needed to establish the prevalence and incidence of CC in various populations, particularly those who are at high risk, like postmenopausal women, diabetics, and those with atypical anginal symptoms. The current evidence mainly comes from single case reports and small case series and is therefore limited by restricted generalizability. Larger studies would more accurately establish the actual clinical burden of the condition and further determine significant demographic or clinical predictors.

Second, future research should concentrate on placing an explicit diagnostic criterion for CC. Currently, diagnosis is determined retrospectively using symptom resolution after intervention in the heart. Having a framework for diagnosis with clinical red flags, such as exertional headache, rapid improvement with nitrates, and lack of neurological findings, would enable physicians to identify the condition earlier and differentiate it from primary headache disorders.

Third, further neuroradiological research will be necessary to elucidate the pathophysiological correlation between headache and myocardial ischemia. Utilizing functional imaging modalities (i.e., PET, fMRI, and SPECT), alterations in cerebral hemodynamics that accompany an ischemic insult can become detectable, enabling the identification of potential biomarkers for cardiac headache-related diseases. Fourth, clinical practice guidelines need to be revised to include the consideration of headache as an anginal equivalent, especially in patients without typical chest pain. This has important implications for emergency medicine, neurology, and primary care, where such patients are often initially mismanaged. Formal educational sessions and interprofessional education can help bridge this knowledge gap and ensure more prompt cardiac assessment in unusual presentations.

Lastly, future studies need to determine the impact of early cardiac screening in patients presenting with exertional or chronic headache, more importantly, those with established cardiovascular risk factors. RCTs with results for patients undergoing early cardiac workups versus standard neurological assessment would provide evidence for practice change. We also recommended that future studies outline clear endpoints, such as the time to diagnosis, the resolution of symptoms following cardiac intervention, the rate of misdiagnosis, and the cost-effectiveness of proposed diagnostic workflows.

## Conclusions

CC is still a rare and underrecognized clinical condition that may masquerade as primary headache syndromes, resulting in delays in diagnosis. Although short-term resolution of symptoms with cardiac interventions, such as nitrate administration or PCI, is frequent, future studies need to separate this from long-term prognostic implications and risk modification. There is an urgent need to design future cohort studies and RCTs for the evaluation of early diagnostic approaches, including the use of red-flag checklists (e.g., exertional headache, cardiovascular risk factors, and focal neurology), cardiac biomarker profiling, and imaging techniques. Potentially useful surrogate markers such as troponin kinetics, red blood cell distribution width, or cerebral hypoperfusion patterns detected using perfusion imaging are promising but require validation.

Contemporary clinical practice guidelines do not adequately cover atypical anginal presentations, including CC. A revision is needed to include interdisciplinary diagnostic paradigms and systematic referral pathways, particularly between cardiology, neurology, and emergency medicine. Comorbid conditions, such as migraine, anxiety, and other chronic headache disorders, need to be taken into account in both the clinical assessment and research design in the future to avoid diagnostic overlap. Teaching programs directed at frontline specialties, such as emergency medicine, neurology, and cardiology, are essential for raising awareness of CC. Standardization of protocols and the application of diagnostic algorithms will simplify clinical practice and enhance patient outcomes.
